# Intravenous Iron Treatment in the Prevention of Iron Deficiency and Anaemia After Roux-en-Y Gastric Bypass

**DOI:** 10.1007/s11695-020-04396-5

**Published:** 2020-01-18

**Authors:** Jorunn Sandvik, Torstein Hole, Christian A. Klöckner, Bård Eirik Kulseng, Arne Wibe

**Affiliations:** 1 Clinic of Medicine and Rehabilitation, Møre and Romsdal Hospital Trust, Alesund, Norway; 2 Center for Obesity, Department of Surgery, St. Olav Hospital, Trondheim University Hospital, Trondheim, Norway; 3 Department of Clinical and Molecular Medicine, Obesity Research Group, Norwegian University of Science and Technology, Trondheim, Norway; 4 Faculty of Medicine and Health Sciences, Norwegian University of Science and Technology, Trondheim, Norway; 5 Department of Psychology, NTNU - Norwegian University of Science and Technology, Trondheim, Norway; 6 Department of Clinical and Molecular Medicine, Norwegian University of Science and Technology, Trondheim, Norway; 7 Department of Surgery, St. Olav Hospital, Trondheim University Hospital, Trondheim, Norway

**Keywords:** Iron deficiency, Anaemia, Intravenous iron replacement, RYGB, Gastric bypass, Bariatric surgery, Iron deficiency anaemia, Iron deficiency without anaemia, Iron deficiency after RYGB

## Abstract

**Background:**

Iron absorption is disturbed after Roux-en-Y gastric bypass (RYGB) and iron deficiency with or without anaemia affects almost half of all patients. Intravenous iron is an option when per oral iron is insufficient or not tolerated. This study explores whether routinely offering intravenous iron treatment when iron stores are empty can prevent anaemia and iron deficiency after RYGB.

**Methods:**

This is a study of prospectively registered data on clinical information, haematological tests and intravenous iron treatment from 644 RYGB patients who underwent surgery between 2004 and 2013, postoperatively followed more than 5 years. Intravenous iron treatment was offered to patients with ferritin ≤ 15 μg/L.

**Results:**

Clinical information was available for all patients at baseline and for 553/644 patients at 5 years; laboratory results were available for 540/644 patients at baseline and 411/644 patients after 5 years. The mean age was 39.8 (± 9.7) years. Overall, 187/483 (38.7%) women and 9/161 (5.6%) men were given intravenous iron treatment in the observation period. From baseline to 5 years, mean haemoglobin decreased by 0.3 g/dL in both men and women. Anaemia occurred in 18/311 (5.8%) women and 9/100 (9%) men at 5 years. Depleted iron stores (ferritin ≤ 15 μg/L) were seen among 44/323(13.6%) women and 3/102 (2.9%) men, and low iron stores (ferritin 16–50 μg/L) occurred in 144/326 (44.6%) women and 38/102 (37.3%) men 5 years after RYGB.

**Conclusion:**

By routinely offering intravenous iron treatment to patients with depleted iron stores after RYGB, haemoglobin levels were preserved. Half of the patients experienced low or depleted iron stores at 5 years.

## Introduction

Roux-en-Y gastric bypass (RYGB) has been a common bariatric procedure for more than 50 years [1], and more than 30% of the 635,000 patients worldwide undergoing a bariatric procedure each year get RYGB [2]. This procedure implies that the food bolus bypasses the main part of the stomach, the duodenum and the proximal jejunum, and instead passes through an alimentary limb of 1 m or more before the food blends with bile and pancreatic enzymes. The aim of this procedure is to reduce capacity for food intake and absorption of energy from the food. The downside is that the main sites for absorption of essential vitamins and minerals, like folate, vitamin B_12_, calcium and iron, are also bypassed, and nutritional elements dependent on acidic environments are less likely to be absorbed [3]. Patients are advised to use supplements of micronutrients to compensate for the reduced absorption and regularly monitor their levels of micronutrients by blood tests [4, 5].

Anaemia has been reported in up to half of the patients 5 years after RYGB, and iron deficiency is even more frequent, due to a combination of lack of intake of iron-rich food and lack of iron absorption [6–8]. Most of the iron used by the cells is recycled, but a daily iron uptake of 1–2 mg is needed to replace the iron losses caused by shedding of epithelial cells from the skin and intestines, menstrual blood and sweat. Symptoms of anaemia are well known, but there is less awareness of symptoms of iron deficiency with normal haemoglobin levels [9].

In addition to haematopoiesis, iron is an essential component of myoglobin in the muscles, necessary for optimal function of neurons, and it is an important element of mitochondrial activity and energy production [10–13]. Iron deficiency without anaemia is a potential cause of fatigue, decreased exercise performance and cognitive impairment, symptoms which are frequently reported after bariatric surgery [14–20].

Iron homeostasis differs from other minerals by having a complex regulation of absorption, recycling and storage, but no mechanism for excretion of surplus iron, and iron overload is toxic [17, 21]. Iron is mainly absorbed by the enterocytes in the duodenum and proximal jejunum, areas that are bypassed after RYGB, but small amounts of iron can be absorbed by the more distal parts of the gastrointestinal tract [22]. The hepatic hormone hepcidin is central in regulating iron absorption from the intestinal lumen to the enterocytes, as well as the transfer of iron from the enterocytes to the blood [23, 24]. When hepcidin levels are high, iron absorption is low. High doses of per oral iron supplements can increase hepcidin and thereby block iron absorption [25, 26].

In clinical settings, iron stores are best assessed by serum ferritin levels [27]. Ferritin plays a major role in iron sequestration and transport, and low ferritin levels are diagnostic for iron deficiency [28]. However, ferritin levels are increased by inflammation, and iron deficiency can therefore coexist with high ferritin levels [29]. The cut-off level for depleted iron stores recommended by WHO is < 15 μg/L for adults [30]. However, higher and lower gender-specific thresholds for ferritin have been used. A ferritin level of 30 μg/L is the most sensitive (92%) and specific (98%) cut-off level for absolute iron deficiency [31]. Haemoglobin levels will remain normal until the iron stores are depleted, and normal haemoglobin levels do not exclude empty iron stores [32].

International guidelines recommend oral iron supplement to prevent iron deficiency and anaemia after bariatric surgery, and intravenous iron treatment if per oral treatment fails [4]. If the iron stores are emptied, it may take several months to restore them by per oral iron supplements [7]. Intravenous iron treatment is a safe procedure that restores iron stores in less time, but access to this treatment can be limited due to financial and organizational causes [9, 33, 34]. In studies published thus far, the indications for intravenous iron treatment after bariatric surgery have been anaemia or per oral iron treatment failure, and less than 10% of the patients have received this treatment after RYGB [6, 35, 36]. The same studies report anaemia in until 30% of the patients. The aim of this study was to explore whether routinely offering intravenous iron treatment to patients with depleted iron stores after RYGB, regardless of haemoglobin levels, preserved iron stores and prevented iron deficiency anaemia 5 years after surgery.

## Material

This study is a retrospective analysis of prospectively collected data on 644 patients who underwent RYGB as a primary treatment for severe obesity at a public hospital from 2004 to 2013 with a postoperative follow-up of more than 5 years.

The RYGB procedure was performed with laparoscopic antecolic, antegastric technique, using a biliopancreatic limb of 40–60 cm and an alimentary limb of 100 cm or 150 cm, depending on BMI below or above 50 kg/m^2^ [37].

The patients were enrolled in a local quality registry and followed a standardized clinical pathway according to international guidelines at the time [38]. A standardized set of lab tests was taken 1 year before the RYGB procedure and at 2, 6, 12, 18, 24, 36, 48 and 60 months after the operation.

Data on weight, comorbidity, complications and other relevant events in the postoperative period were registered. In addition to information from planned visits in the follow-up program of 5 years, information on intravenous iron treatment was recorded until 14 years after RYGB. Results from laboratory tests related to the 5-year outpatient follow-up program were added to the quality registry by collecting data directly from the hospital’s laboratory data system. The registry was last updated January 2019. Laboratory results were available in the registry for 544 (84%) patients at baseline and 428 (66%) after 5 years.

The over-the-counter multivitamin-mineral product most commonly used in this cohort contained 15 mg iron (II) fumarate and 400 μg folate per unit. In general, the patients were advised to use additional per oral iron supplements with ascorbic acid for 1 month twice a year if ferritin was > 50 μg/L, or more often if the ferritin values were lower. Patients with ferritin levels above the normal range were advised not to take iron supplements. The patients were also recommended supplemental vitamin B_12_ and calcium with vitamin D, and they were offered intravenous iron treatment if the iron stores were depleted, defined as ferritin ≤ 15 μg/L.

## Methods

Continuous variables are given as means ± standard deviation (SD) if normally distributed, and median with interquartile range (IQR) in non-normally distributed variables. Categorical variables are reported in numbers and percentages. Independent *t* tests were performed for normally distributed variables, and non-parametric tests were used for non-normally distributed variables. *χ*^2^ tests were performed for categorical variables. Differences were considered to be significant at *p* < 0.05.

Iron stores were graded as depleted (ferritin ≤ 15 μg/L), low (ferritin 16–50 μg/L), moderate (ferritin 51–100 μg/L), and replete (ferritin > 100 μg/L). Intravenous iron treatment was given mainly as ferric carboxymaltose 1 g in one visit and less often as iron sucrose 200 mg over five visits. Blood transfusions for iron deficiency anaemia were not given unless there was an acute medical situation, like haemorrhage after elective or acute surgery, in the observation period.

Statistical analyses were performed using IBM SPSS version 25 (SPSS Inc., Chicago, IL, USA) and STATA 14 (StataCorp).

## Results

A total of 644 patients underwent RYGB as a primary bariatric surgery for severe obesity from 2004 to 2013. Mean (SD) age was 39.8 ± 9.7 years, and 75% of the patients were women. In the observation period of 5 to 14 years, mean 112 ± 29.3 months, fifteen (2.3%) patients have died, two of them in the early postoperative period. Baseline body mass index (BMI) was 43.9 ± 5.1 kg/m^2^ and percentage total weight loss (%TWL) at 5 years was 27.6 ± 10.1 kg/m^2^ (Table 1).

**Table 1:** Patients’ characteristics

	All, *N* = 644	Female, *n* = 483	Male, *n* = 161
Age*(years)	39.8 ± 9.7; 644	39.4 ± 9.6; 483	40.9 ± 10.1; 161
BMI baseline* (kg/m^2^)	43.9 ± 5.1; 644	43.8 ± 4.8; 483	44.3 ± 5.7; 161
BMI nadir* (kg/m^2^)	28.5 ± 4.3; 633	28.0 ± 4.1; 474	29.7 ± 4.3; 159
BMI 5 years* (kg/m^2^)	31.6 ± 5.3; 553	31.2 ± 5.5; 418	32.6 ± 4.5; 135
%TWL nadir* (%)	35.3 ± 7.8; 633	36.1 ± 7.5; 474	32.8 ± 8.0; 159
%TWL 5 years* (%)	27.6 ± 10.1; 533	28.3 ± 10.2; 418	25.2 ± 9.3; 135
%EWL 5 years* (%)	65.8 ± 24.4; 533	67.8 ± 25.0; 418	59.9 ± 21.6; 135

*Mean ± SD, *BMI* body mass index, *%TWL* percentage total weight loss, *%EWL* percentage excess weight loss

Median (IQR) ferritin changed from 61 (36–100) μg/L to 43 (23–69) μg/L in women, and from 173 (123–265) μg/L to 62 (40–93) μg/L in men before to 5 years after RYGB (Table 2).

**Table 2:** Haemoglobin (Hgb) and serum ferritin levels yearly from before to 5 years after RYGB

	Baseline	Operation	1 year	2 years	3 years	4 years	5 years
Hgb g/dL (mean ± SD); all patients	14.1 ± 1.2; 539/644	14.3 ± 1.2; 609/644	13.6 ± 1.1; 542/644	13.5 ± 1.2; 504/644	13.5 ± 1.2; 481/644	13.6 ± 1.2; 462/644	13.8 ± 1.2; 411/644
Hgb g/dL (mean ± SD); women	13.7 ± 1.0; 398/483	13.9 ± 1.0; 451/483	13.3 ± 1.0; 401/483	13.2 ± 1.0; 382/483	13.2 ± 1.0; 370/483	13.3 ± 1.0; 353/483	13.4 ± 1.0; 311/483
Hgb g/dL (mean ± SD); men	15.2 ± 0.9; 141/161	15.3 ± 1.0; 158/161	14.5 ± 1.0; 141/161	14.7 ± 1.0; 122/161	14.6 ± 0.9; 111/161	14.7 ± 1.0; 109/161	14.9 ± 0.9; 100/161
Ferritin μg/L; median (IQR); all patients	80 (42–141); 544/644	111 (60–211); 282/644	76 (33–133); 542/644	59 (31–105); 504/644	45 (22–84); 488/644	43 (23–81); 472/644	47 (25–75; 428/644
Ferritin μg/L; median (IQR); women	61 (36–100); 401/483	96 (49–149); 214/483	59 (27–104); 405/483	50 (26–89); 382/483	40 (21–74); 375/483	36 (21–70); 361/483	43 (23–69); 326/483
Ferritin μg/L; median (IQR); men	173 (123–265); 143/161	236 (161–319); 68/161	135 (91–205); 137/161	101 (68–174); 122/161	78 (48–122); 113/161	75 (42–120); 111/161	62 (40–93); 102/161

**Fig. 1:**
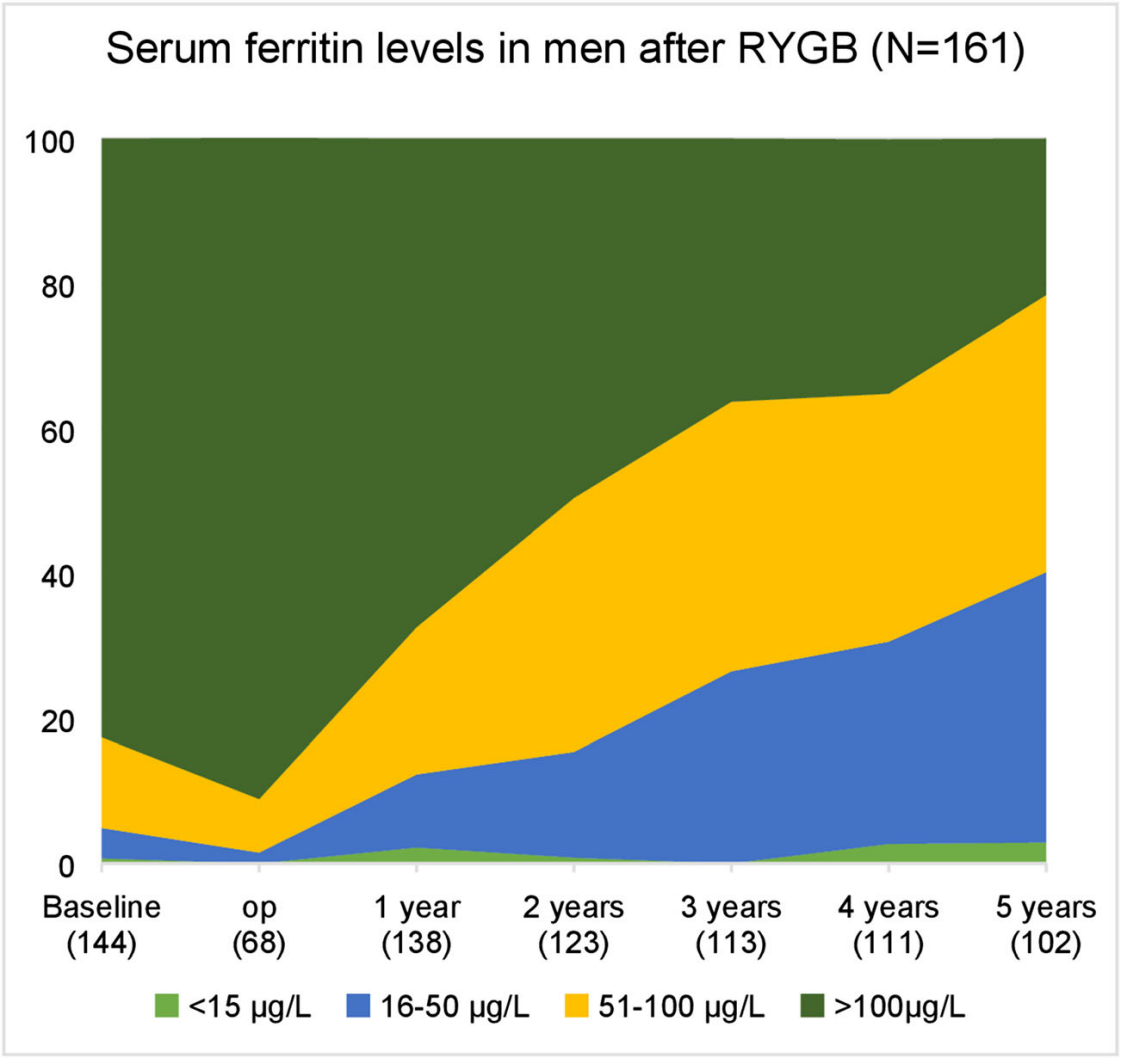
Serum ferritin from before to 5 years after RYGB in men

**Fig. 2:**
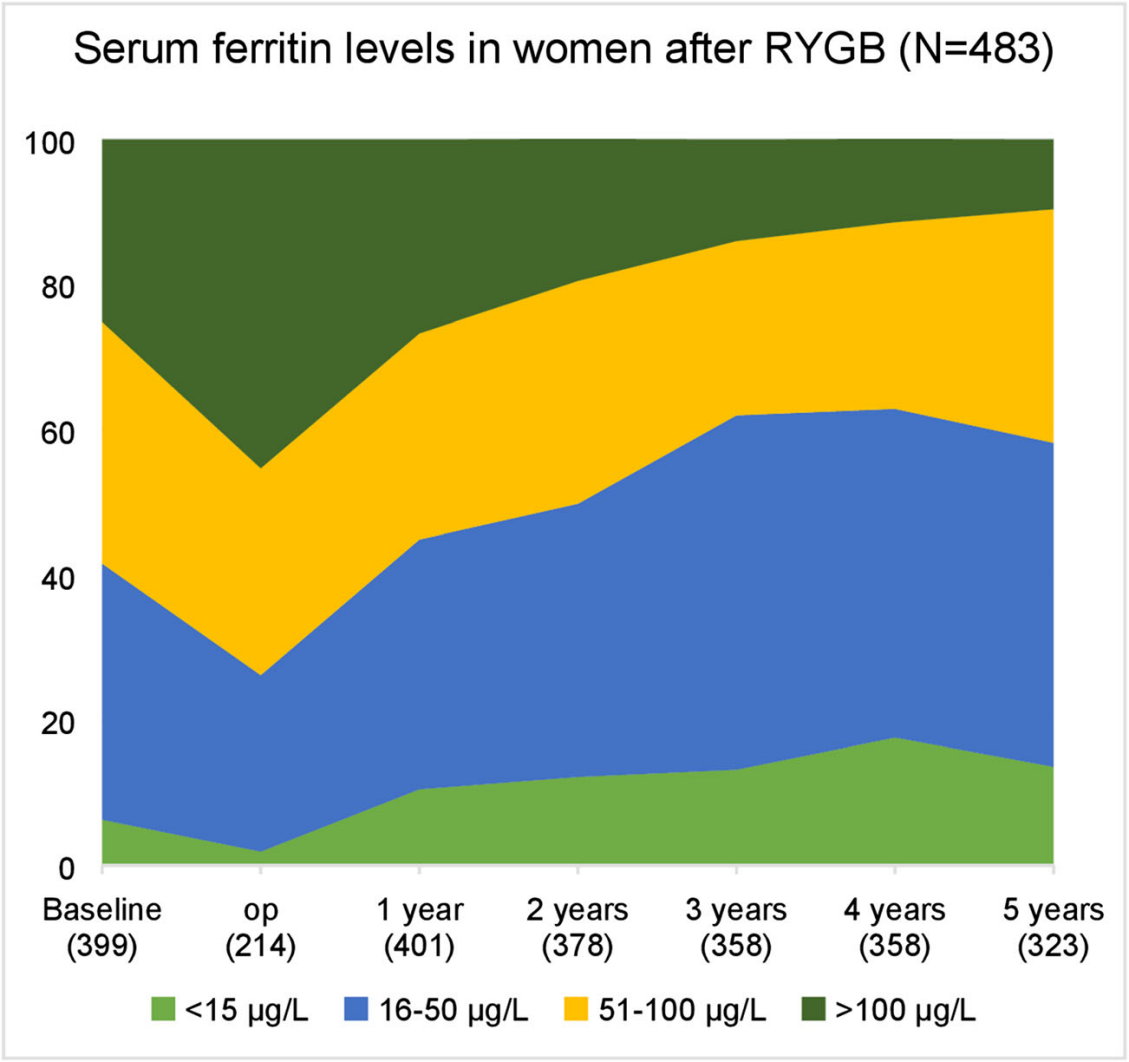
Serum ferritin from baseline to 5 years after RYGB in women

**Fig. 3:**
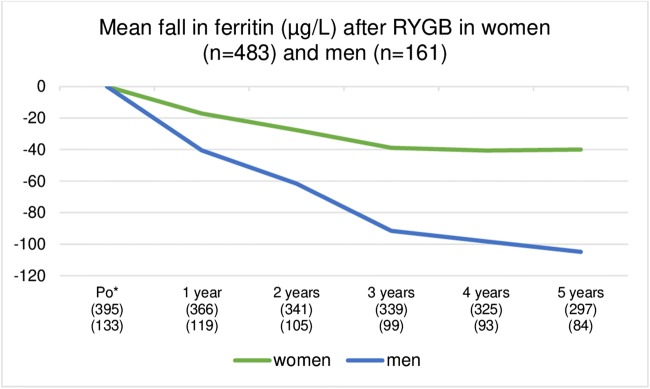
Mean change in ferritin (μg/L) after RYGB. Asterisk means ferritin before RYGB might be increased due to obesity induced inflammation; the ferritin value 1–2 months after RYGB are used as reference

The number of patients experiencing iron depletion increased from 25/399 (6.3%) before RYGB to 44/323 (13.6%) after 5 years among women and from 1/143 (0.7%) to 3/102 (2.9%) among men. The number of patients with low iron stores (ferritin 16–50 μg/L) was 141/399 (35.3%) before RYGB and 144/323 (44.6%) after 5 years among women and 5/143 (3.5%) before and 38/192 (37.3%) after 5 years among men (Figs. 1 and 2). The mean reduction in ferritin values in the 5 years after RYGB was 40 ± 85 μg/L in women and 105 ± 83 μg/L in men (Fig. 3).

In the Scandinavian population, anaemia has been defined as haemoglobin < 12.0 g/dL in women and haemoglobin < 13.7 g/dL in men [39]. According to these definitions, anaemia occurred in 13/451 (2.9%) of women and 7/158 (4.4%) of men at RYGB, in 36/382 (9.4%) of women and 16/122 (13.1%) of men after 2 years, and in 18/311 (5.8%) of women, and 9/100 (9.0%) of men 5 years after RYGB.

**Fig. 4:**
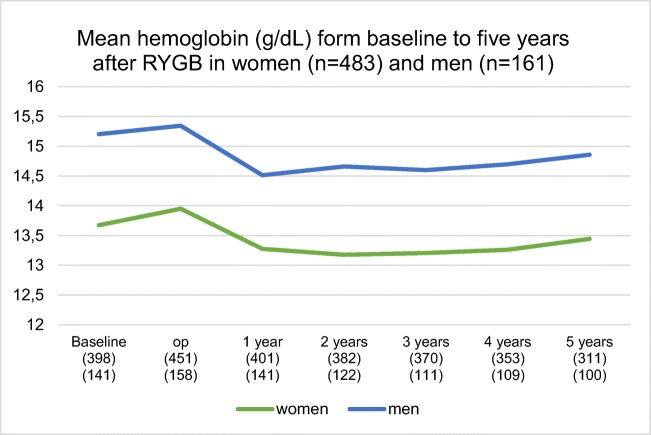
Mean haemoglobin levels from baseline to 5 years after RYGB

Mean haemoglobin levels changed from 13.7 ± 1.0 g/dL before to 13.4 ± 1.0 g/dL 5 years after RYGB in women (*p* < 0.001) and from 15.2 ± 0.95 g/dL to 14.9 ± 0.95 g/dL in men (*p* < 0.001) (Fig. 4). Annual values are given in Table 2.

With a mean observation time of 9.25 ± 2.4 years after RYGB, a total of 196/644 (30.4%) patients, 187/483 (38.7%) women and 9/161 (5.6%) men, had one or more intravenous iron treatments. Median (IQR) time from RYGB to first intravenous iron was 3 (2–4) years for women and 4 (2–7) years for men. Among the patients who were given intravenous iron treatment, 102 (16% of the study population) were given one treatment, 42 (6.6% of the study population) were given two treatments and 27 (4.2% of the study population) were given three treatments in the observation period. Thirty-two patients (5% of the study population) had their first intravenous iron treatment more than 5 years after RYGB.

The levels of folate and vitamin B_12_ increased after RYGB, indicating that most patients were adherent to the recommended supplements.

## Discussion

The main findings in this study are that during the first 5 years after RYGB; more than one out of four patients were given intravenous iron treatment when this treatment was routinely offered to iron depleted patients. There was a minor decrease in mean haemoglobin levels in the group, but a substantial increase in the proportion of patients with low or depleted iron stores 5 years after RYGB.

Compared with other studies, intravenous iron treatment was more widely applied in the present cohort and also given to patients without anaemia if ferritin levels were low [7, 35]. However, half of those who were in need of intravenous iron only had a single treatment over the average observation time of 9 years. This could be due to better compliance to per oral prophylactic treatment, improvement in iron absorption over time, food modifications or treatment for underlying disease after the first treatment with intravenous iron. It is also possible that iron deficiency was less likely to be diagnosed when the follow-up was handed over from the bariatric outpatient clinic to primary health care 5 years after RYGB.

In a systematic review on iron deficiency after RYGB and sleeve gastrectomy with an average follow-up time of 27.8 months, the overall incidence of iron deficiency anaemia were 14.8% post-RYGB, and iron deficiency occurred in 22.5% [40]. Only two of the studies included in the review reported on intravenous iron treatment.

In a retrospective study by Obinwanne, 53% of the RYGB patients were found to have ferritin < 50 μg/L at some point in the postoperative period [6]. In this study, only 6.7% of the patients were given intravenous iron treatment, and mean haemoglobin changed from 13.5 g/dL preoperatively to 11.6 g/dL more than 5 years after RYGB. Compared with the present study, the proportion of patients with ferritin < 50 μg/L is almost at the same level, but the fall in haemoglobin of 1.9 g/dL compared with 0.3 g/dl may be due to a lesser extent of intravenous iron treatment. In another retrospective study where no use of intravenous iron was reported, a quarter of the women were anaemic, and 42% had depleted iron stores 5 years after RYGB [41].

The number of post-bariatric patients is continually increasing, and the capacity at specialized centres for individualized follow-up is limited. However, lifelong follow-up is important to avoid vitamin and mineral deficiencies. For water-soluble vitamins and minerals, general recommendations are feasible. Iron supplement, however, needs to be individualized, and as the regulation of iron uptake is complex, there is a need for educating patients as well as primary health care providers on how to avoid iron deficiency as well as overuse of iron supplements after bariatric surgery.

According to the findings in the present study, access to intravenous iron treatment when iron stores are empty prevents anaemia 5 years after RYGB in women, but not to the same degree in men. To prevent low or depleted iron stores after RYGB, an even more liberal indication for intravenous iron treatment might be necessary.

Iron deficiency even in the absence of anaemia has been related to fatigue and lower ability to respond to increases in mental and physical workload [42]. Fatigue is a major complaint among RYGB patients [19]. In non-anaemic iron-deficient adults, iron supplementation has been associated with reduced fatigue, although without any objective measurements of improved physical capacity [43, 44]. The present study did not contain systematic information on fatigue or other symptoms that could be related to iron deficiency, and any connection between fatigue and low iron stores could not be explored.

### Strengths and Limitations

The strength of this study was the close follow-up of the patients in a standardized post-operative program with haematological results of more than 65% of the patients at each point in time. All treatments with intravenous iron were registered prospectively.

The limitations were the lack of systematic information on symptoms, ferritin and haemoglobin levels before and after each intravenous iron treatment, and that haematological results were not collected for more than 5 years after RYGB. Also, there was no information recorded on menstrual status or to what degree the patients followed the recommendations on per oral iron supplements.

## Conclusion

Iron deficiency and anaemia are common in the long run after RYGB. Individualized advice on iron supplements and access to intravenous iron treatment when iron stores were depleted seemed to reduce the frequency of anaemia, but did not prevent iron deficiency in the present study. Major falls in iron stores appeared more than 2 years after surgery, when many patients are no longer followed in bariatric outpatient clinics. The primary health care providers might not be aware of the need for lifelong individualized iron supplements for RYGB patients. The clinical relevance of low iron stores after RYGB needs further investigation. If there is an association between iron deficiency and fatigue among these patients, better access to intravenous iron treatment can contribute to improve health after RYGB.
